# Does Measles Vaccination Reduce the Risk of Acute Respiratory Infection (ARI) and Diarrhea in Children: A Multi-Country Study?

**DOI:** 10.1371/journal.pone.0169713

**Published:** 2017-01-11

**Authors:** Rahul Bawankule, Abhishek Singh, Kaushalendra Kumar, Sadanand Shetye

**Affiliations:** 1 International Institute for Population Sciences, Mumbai, India; 2 Department of Public Health and Mortality Studies, International Institute for Population Sciences, Mumbai, India; 3 B K L Walawalkar Rural Medical College, Derwan, India; University of North Carolina at Chapel Hill School of Dentistry, UNITED STATES

## Abstract

**Background:**

Pneumonia and diarrhea occur either as complications or secondary infections in measles affected children. So, the integrated Global Action Plan for Pneumonia and Diarrhea (GAPPD) by WHO and UNICEF includes measles vaccination as preventive measure in children. The objective of the study is to examine the effect of measles vaccination on Acute Respiratory Infection (ARI) and diarrhea in children in the Democratic Republic of Congo, Ethiopia, India, Nigeria, and Pakistan.

**Methods:**

We analyzed data from the most recent rounds of Demographic and Health Surveys (DHS) in the selected countries. We included children age 12–59 months in the analysis. We used multivariable binary logistic regression to examine the effect of measles vaccination on ARI and diarrhea in children. We also estimated Vaccination Effectiveness (VE).

**Findings:**

More than 60 percent of the children age 12–59 months were given measles vaccine before the survey in the Democratic Republic of Congo, Ethiopia, India and Pakistan. Children who were given the measles vaccine were less likely to suffer from ARI than unvaccinated children in India and Pakistan. Children who were given the measles vaccine had a lower risk of diarrhea than those who did not receive it in all the selected countries except Ethiopia. Measles vaccination was associated with reduction in ARI cases by 15–30 percent in India and Pakistan, and diarrhea cases by 12–22 percent in the Democratic Republic of Congo, India, Nigeria and Pakistan.

**Conclusion:**

The receipt of the measles vaccine was associated with decrease in ARI and diarrhea in children. The immunization program must ensure that each child gets the recommended doses of measles vaccine at the appropriate age. The measles vaccination should be given more attention as a preventive intervention under the Global Action Plan for Pneumonia and Diarrhea (GAPPD) in all low and middle-income countries.

## Introduction

Measles is a highly contagious disease transmitted through a virus belonging to the Morbillivirus genus. The infection is acquired via the respiratory tract and is mostly seen in infants and children below five years of age [1]. The virus usually disrupts the epithelial cells and suppresses the immune system leading to infection in various organ systems. Immune suppression can last even after recovery and thereby increases susceptibility to secondary infections. The respiratory and intestinal tracts are the most affected sites in measles-infected children. When the measles virus affects the lower respiratory tract epithelium and destroys local immunity within the lungs, an individual suffers from pneumonia [2,3]. Similarly, the protein losing enteropathy in measles can lead to diarrhea [4]. Thus, pneumonia and diarrhea occur in 10–40 percent of measles cases either as complications or as secondary infections [2].

Often, pneumonia and diarrhea increase the risk of death in the measles-infected individual. Over the last few decades, a substantial decline has been reported in the incidence of measles and measles related mortality in many countries. Yet, it remains a significant cause of child mortality globally and has caused about 2 percent of child deaths in 2013 [5]. Estimates suggest that of the 0.12 million measles-associated death in 2012, 43 percent occurred in Southeast Asia and 36 percent in the sub-Saharan Africa. India alone accounted for 14 percent of the deaths. These numbers are striking given that measles is a vaccine-preventable disease and that the vaccine also reduces the susceptibility to pneumonia and diarrhea in children [6].

Pneumonia is the most severe form of ARI affecting the lungs. In comparison, diarrhea is the typical manifestation of gastrointestinal infections caused by a wide range of pathogens [7,8]. Estimates suggest that pneumonia and diarrhea are the leading causes of child mortality [5]. In 2010, pneumonia and diarrhea together accounted for 33 percent of child deaths in Southeast Asia and 29 percent in sub-Saharan Africa [9]. While repeated episodes of pneumonia in children have a long-term effect on the respiratory system, repeated episodes of diarrhea in children lead to malnutrition and diminished neurological and cognitive development [10–13].

It is evident that pneumonia and diarrhea have serious consequences for children. Fortunately, both are avoidable with simple measures [14,15]. Childhood vaccination is one of the safest measures to prevent pneumonia and diarrhea in children [16,17]. Among available vaccines, measles vaccine protects against both measles and its related complications. Besides, the indirect benefit of measles vaccination like other childhood vaccines includes making children healthier and increasing their capability to combat other pathogens causing ARI and diarrhea. Hence, in countries where measles is a significant cause of child mortality, measles vaccination can reduce the incidence of pneumonia and diarrhea and the associated mortality [18]. In response, WHO and UNICEF have introduced measles vaccination as a preventive measure in the integrated Global Action Plan for Pneumonia and Diarrhea (GAPPD)[19].

Though measles vaccination has been efficacious in preventing pneumonia and diarrhea in children, no study so far, has examined the occurrence of pneumonia and diarrhea in measles-vaccinated children or compared the occurrence of pneumonia and diarrhea in measles-vaccinated children with that in measles-unvaccinated children. Only a few studies have identified the lack of measles vaccination as a predictor of pneumonia and diarrhea [20–24]. These studies have obvious limitations as they are small-scale studies that lack generalizability. This study aims to evaluate the effectiveness of measles vaccination on ARI and diarrhea in the Democratic Republic of Congo, Ethiopia, India, Nigeria and Pakistan. These countries were selected for the analysis because they have the highest population of measles-unvaccinated children and the highest concentration of child deaths associated with pneumonia and diarrhea [9,25].

## Methods

### Ethical Approval

Our study is based on the Demographic and Health Survey (DHS). The data is readily available on the DHS website http://dhsprogram.com/data/available-datasets.cfm and can be accessed for research with prior permission. It does not have any identifiable information on the survey participants. DHS strictly follows all the ethical concerns, including informed consent, hence no ethical approval or informed consent was required for the current study.

### Data Source

We used data from the most recent rounds of Demographic and Health Survey (DHS) conducted in the five selected countries. DHSs are nationally representative population-based cross-sectional surveys being carried out in developing countries since 1984. They attempt to collect and disseminate accurate data on fertility, family planning, maternal and child health, gender, HIV/AIDS, malaria, and nutrition. DHS uses multistage sampling design, standardized questionnaires and field procedures for data collection. Interviews were conducted with 18827, 16515, 124385, 38948, and 13558 women in the Democratic Republic of Congo, Ethiopia, India, Nigeria and Pakistan, respectively. The response rate was above 90 percent in all the five countries. The information related to child health is collected from the mother. More methodologic details about these surveys can be obtained from the country specific DHS reports [26–31].

### Inclusion and Exclusion Criteria

Since the measles vaccine is given between 9 and 12 months of age, we excluded those children who had not completed 12 months of age at the time of the survey. DHS collects information on vaccination status for up to three last births in five years preceding the survey i.e. youngest, next and second from youngest children. Secondly, the child is considered to be vaccinated if vaccination dates are marked on the vaccination card. In absence of the vaccination card reporting by the mother is recorded. The reporting of vaccination status by the mother was higher for younger and second younger children. Hence, we confined our analysis only to the youngest child born in the reference time of the respective surveys. As a result, the analysis presented in the subsequent sections of the paper is based on the youngest child aged 12–59 months. The analytical sample sizes in the Democratic Republic of Congo, Ethiopia, India, Nigeria and Pakistan are 7406, 5540, 27354, 14073 and 5249 respectively.

### Exposure Variable

The primary exposure variable is measles vaccination. It is coded as ‘1’ if the child has received an injection to prevent measles. It is coded as ‘0’ if the child has not received an injection to prevent measles.

### Outcome Variable

The two outcome variables included in the analysis are occurrence of ARI and diarrhea. A child was considered suffering from ARI if s/he had a cough with short and rapid breathing associated with the problem in the chest during the fifteen days preceding the survey. Similarly, a child was considered suffering from diarrhea if s/he had a diarrheal episode during the two weeks preceding the survey. Both the outcome variables are binary variables where ‘0’ refers to not suffered from illness and ‘1’ refers to suffered from illness.

### Control Variables

We identified potential socioeconomic and demographic risk factors of ARI and diarrhea and included those as control variables in the analysis [22,32–40]. The common control variables for both illnesses are age of the child, sex and birth size of the child, mother’s literacy and her exposure to mass media, religion, caste/tribe, wealth status and place of residence. We also included ‘type of cooking fuel’ as a risk factor for ARI, and ‘type of toilet facility’ and ‘source of drinking water ‘as risk factors for diarrhea.

In DHS, the household wealth index is computed using principal components analysis assigning weights to country specific assets. The assets considered for the construction of wealth index include the type of cooking fuel, toilet facility and source of drinking water. Since, we wanted to examine the independent effect of ‘type of cooking fuel’ on ARI and the effect of ‘type of toilet facility’ and ‘source of drinking water’ on diarrhea, we generated a new wealth index which did not include type of cooking fuel, type of toilet facility and the source of drinking water. We followed the procedure of DHS to generate a new country specific wealth index. Finally, we categorized the households in each country into the lowest one-third (poor), middle one-third (middle) and the highest one-third (rich).

The variable on media exposure includes exposure to newspaper, television and radio. The mothers who were exposed to any source of media were coded as ‘exposed’. The rest were coded as ‘not exposed’. Following DHS classification, we grouped the type of cooking fuels into ‘solid’ and ‘other’ [26–31]. Further, the toilet facility and source of drinking water were categorized into ‘improved’ and ‘unimproved’ following the WHO/UNICEF definition [41]. The details of the control variables are given in S1 Appendix.

### Statistical Analysis

We calculated the mean age of both measles vaccinated and unvaccinated children in all countries. Bivariate and multivariate analysis were carried out to fulfill the objectives of the paper. We estimated the prevalence of measles vaccination, ARI and diarrhea in children age 12–59 months. We used two separate multivariable binary logistic regression models to examine the effect of measles vaccination on ARI and diarrhea in children. Having established a significant statistical relationship between lack of measles vaccination and occurrence of ARI and diarrhea, we further estimated the vaccination effectiveness (VE) [42,43].

The VE is given by,

VE=100×(1−AOR)

where, AOR is the adjusted odds ratio of ARI/diarrhea in children who did not receive the measles vaccine.

We used STATA 13.0 to carry out the analysis.

## Results

**Fig 1:**
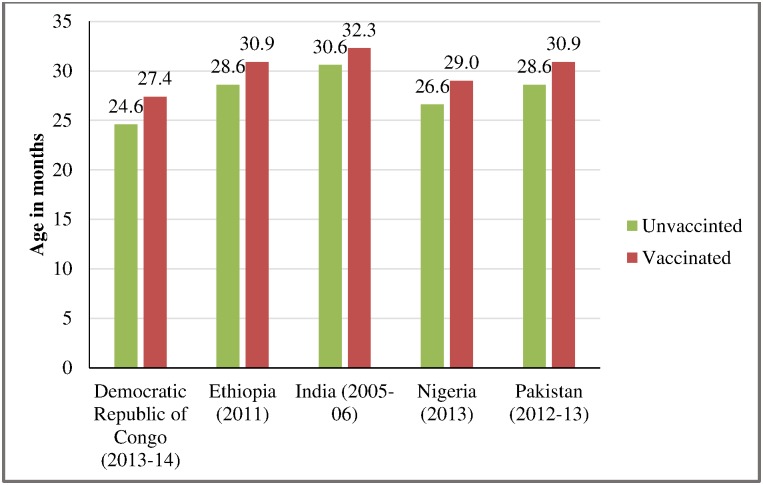
Mean age of measles vaccinated and unvaccinated children age 12–59 months in selected countries.

The mean age of measles vaccinated children was generally higher than mean age of unvaccinated children in all countries. It ranged between 27 months in the Democratic Republic of Congo and 32 months in India. Similarly, the lowest and highest mean ages of unvaccinated children were 25 months in the Democratic Republic of Congo and 30 months in India (Fig 1).

**Fig 2:**
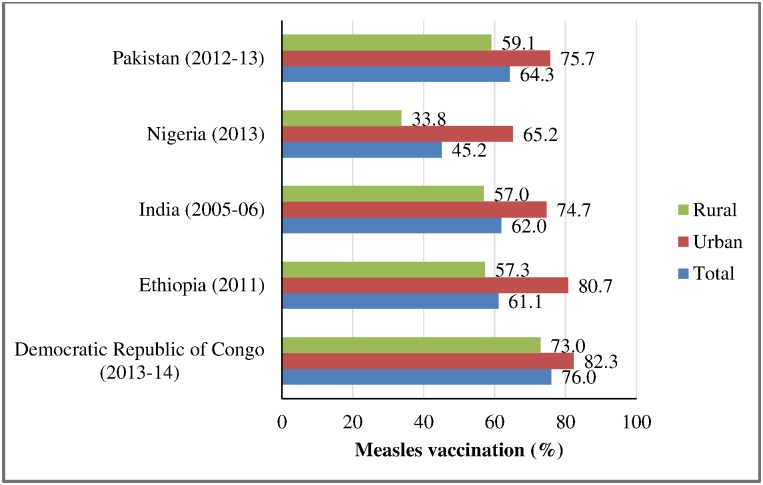
Coverage of measles vaccination in children age 12–59 months in selected countries.

The coverage of measles vaccination was more than 60 percent in children age 12–59 months in all the selected countries except Nigeria. Overall, 76 percent of the children in the Democratic Republic of Congo, 61 percent in Ethiopia, 62 percent in India, 45 percent in Nigeria and 64 percent in Pakistan had received measles vaccination. The coverage of measles vaccination was generally higher in urban than in rural areas (Fig 2).

**Fig 3:**
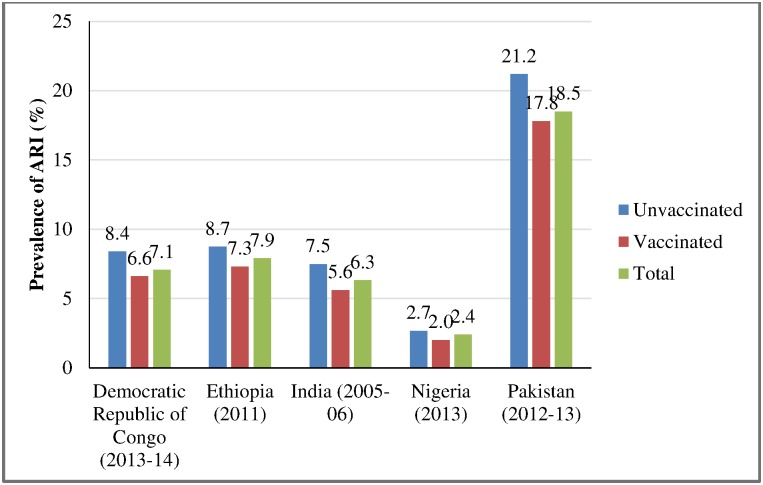
Prevalence of ARI in children age 12–59 months in selected countries by measles vaccination.

The prevalence of ARI in two-weeks preceding the survey ranged between 2 percent and 18 percent. The lowest and highest prevalence of ARI were in Nigeria and Pakistan respectively. The prevalence of ARI varied considerably by the measles vaccination status in the Democratic Republic of Congo, Ethiopia, India and Pakistan. The prevalence of ARI was lower in measles-vaccinated children compared to measles unvaccinated children (Fig 3).

**Fig 4:**
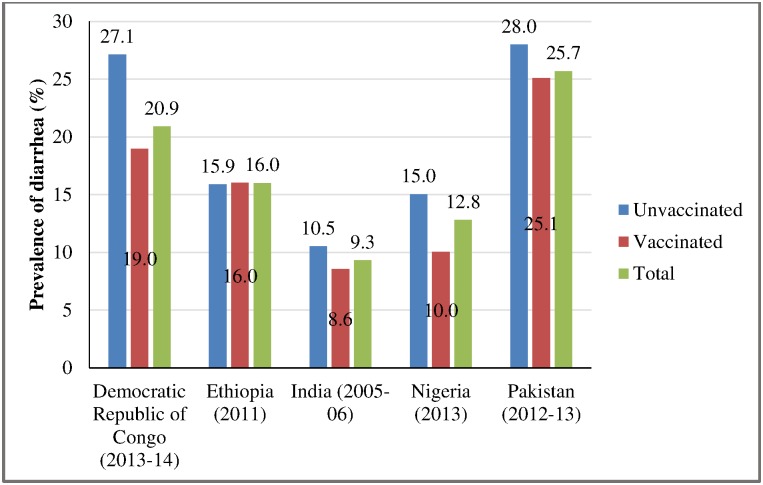
Prevalence of diarrhea in children age 12–59 months in selected countries by measles vaccination.

The prevalence of diarrhea in two-weeks preceding the survey also varied considerably across the selected countries. It ranged between 9 percent (India) and 26 percent (Pakistan) and was considerably high in the Democratic Republic of Congo (21%). The prevalence of diarrhea also varied by the measles vaccination status in the Democratic Republic of Congo, India, Nigeria and Pakistan but not in Ethiopia (Fig 4).

The prevalence of ARI by socioeconomic, demographic and residence related variables is shown in Table 1. The prevalence of ARI was higher in children belonging to households using solid cooking fuel and those in the age group 12–35 months. ARI did not vary by the sex of the child in all the selected countries. The prevalence of ARI did not vary by the size of child, mother’s literacy and religion in Nigeria. The prevalence of ARI was marginally higher in children of mothers who were not exposed to mass media in India, Nigeria and Pakistan and was highest in children belonging to the poor wealth status in the Democratic Republic of Congo, India and Nigeria.

**Table 1:** Prevalence^1^ of ARI in children age 12–59 months by socioeconomic, demographic and residence related variables in selected countries.

Socioeconomic, demographic and residence related characteristics	Prevalence (95% CI)				
	Democratic Republic of Congo	Ethiopia	India	Nigeria	Pakistan
	2013–14	2011	2005–06	2013	2012–13
	n = 6996	n = 5185	n = 26038	n = 12822	n = 4954
**Measles vaccination**					
Unvaccinated	8.4 (6.8–10.5)	8.7 (7.0–10.9)	7.5 (6.7–8.4)	2.7 (2.2–3.3)	21.2 (17.7–25.1)
Vaccinated	6.6 (5.6–7.9)	7.3 (6.1–8.8)	5.6 (5.1–6.2)	2.0 (1.6–2.5)	17.8 (15.7–20.2)
**Type of cooking fuel**					
Solid	7.2 (6.1–8.3)	7.9 (6.8–9.2)	6.9 (6.3–7.5)	2.7 (2.3–3.2)	18.7 (16.4–21.4)
Other	1.4 (0.2–9.9)	6.3 (2.0–18.6)	4.3 (3.6–5.0)	1.1 (0.7–1.7)	18.0 (14.6–22.0)
**Age of child (in months)**					
12–35	8.1 (6.8–9.5)	8.8 (7.4–10.4)	6.8 (6.2–7.5)	2.9 (2.4–3.4)	19.8 (17.4–22.5)
36–59	3.7 (2.7–5.1)	6.7 (5.0–8.8)	5.5 (4.9–6.2)	1.2 (0.8–1.7)	15.7 (13.1–18.7)
**Sex of child**					
Male	7.1 (5.9–8.7)	8.2 (6.7–10.0)	6.4 (5.8–7.0)	2.3 (1.9–2.9)	19.0 (16.6–21.7)
Female	7.0 (5.7–8.6)	7.5 (6.0–9.3)	6.2 (5.6–6.9)	2.4 (2.0–2.9)	17.9 (15.4–20.7)
**Birth size of child**					
Average or larger	7.1 (6.0–8.3)	7.1 (5.9–8.5)	6.0 (5.5–6.6)	2.3 (2.0–2.7)	18.1 (16.0–20.4)
Smaller than average	6.9 (4.9–9.7)	9.7 (7.6–12.2)	7.3 (6.4–8.4)	2.6 (2.0–3.6)	20.0 (16.4–24.2)
**Literacy status of mother**					
Illiterate	8.4 (5.9–11.8)	8.7 (7.3–10.4)	6.8 (6.0–7.6)	2.4 (1.9–3.1)	19.1 (16.5–22.1)
Literate	6.8 (5.8–7.9)	6.1 (4.5–8.1)	5.9 (5.4–6.5)	2.3 (1.9–2.8)	17.7 (15.2–20.4)
**Mother exposed to mass media**					
Not exposed	6.7 (5.5–8.2)	7.2 (5.7–9.0)	7.3 (6.4–8.3)	2.9 (2.3–3.7)	19.1 (16.0–22.7)
Exposed	7.5 (6.1–9.1)	8.3 (6.9–10.0)	5.9 (5.4–6.4)	2.1 (1.7–2.5)	18.2 (16.0–20.7)
**Religion**^2^					
Main	7.6 (6.2–9.3)	7.7 (6.5–9.2)	5.7 (5.2–6.2)	2.4 (1.9–3.0)	NC
Other	6.4 (5.2–7.8)	8.2 (6.2–10.8)	8.6 (7.5–9.9)	2.3 (1.9–2.9)	NC
**Caste/Tribe**					
Scheduled Caste	NA	NA	6.2 (5.3–7.2)	NA	NA
Scheduled Tribe	NA	NA	4.2 (3.3–5.4)	NA	NA
Other	NA	NA	6.4 (5.8–7.0)	NA	NA
**Wealth status**					
Poor	7.6 (6.0–9.5)	7.8 (6.0–10.1)	7.3 (6.5–8.1)	3.1 (2.5–3.8)	18.3 (15.5–21.6)
Middle	7.5 (5.9–9.4)	9.4 (7.7–11.6)	6.0 (5.3–6.8)	2.4 (1.9–3.2)	20.7 (17.4–24.5)
Rich	6.4 (5.0–8.1)	4.3 (2.9–6.2)	4.6 (4.0–5.4)	1.4 (1.0–1.9)	16.1 (13.5–19.1)
**Place of residence**					
Urban	6.2 (4.8–7.9)	4.6 (2.7–7.6)	5.3 (4.6–6.2)	1.9 (1.4–2.4)	17.5 (14.2–21.3)
Rural	7.5 (6.2–9.1)	8.5 (7.3–9.9)	6.7 (6.1–7.3)	2.7 (2.2–3.2)	18.9 (16.5–21.7)
**Total**	**7.1 (6.0–8.2)**	**7.9 (6.8–9.1)**	**6.3 (5.8–6.8)**	**2.4 (2.0–2.8)**	**18.5 (16.5–20.7)**

Abbreviations: CI, confidence interval; NA, Not applicable, NC: not collected.

All values are weighted.

^1^ estimated per 100 children.

^2^ termed as main if followed in the majority of households in the particular country (Christian in Democratic Republic of Congo and Ethiopia, Hindu in India and Islam in Nigeria).

Table 2 shows the prevalence of diarrhea in children age 12–59 months by socioeconomic, demographic and residence related variables. The prevalence of diarrhea in children varied by the type of toilet facility in the Democratic Republic of Congo, Ethiopia, India and Pakistan. The source of drinking water was associated with diarrhea only in Pakistan. The prevalence of diarrhea was higher in male children and children age 12–35 months in the Democratic Republic of Congo, Ethiopia, and India. The prevalence of diarrhea was highest in children belonging to poor households in the Democratic Republic of Congo, Ethiopia, India and Nigeria.

**Table 2:** Prevalence^1^ of diarrhea in children age 12–59 months by socioeconomic, demographic and residence related variables in selected countries.

Socioeconomic, demographic and residence related characteristics	Prevalence (95% CI)				
	Democratic Republic of Congo	Ethiopia	India	Nigeria	Pakistan
	2013–14	2011	2005–06	2013	2012–13
	n = 6999	n = 5191	n = 26065	n = 12878	n = 4971
**Measles vaccination**					
Unvaccinated	27.1 (24.0–30.5)	15.9 (13.3–18.8)	10.5 (9.6–11.5)	15.0 (13.6–16.6)	28.0 (24.1–32.3)
Vaccinated	19.0 (17.2–20.9)	16.0 (14.2–18.0)	8.6 (8.0–9.2)	10.0 (8.9–11.2)	25.1 (23.0–27.3)
**Type of toilet facility**					
Improved and not shared	18.1 (15.3–21.2)	13.9 (10.0–19.1)	8.1 (7.3–9.0)	12.6 (11.1–14.2)	24.0 (21.9–26.3)
Unimproved	21.6 (19.8–23.5)	16.2 (14.4–18.0)	9.7 (9.1–10.4)	12.9 (11.7–14.1)	27.6 (24.7–30.7)
**Source of drinking water**					
Improved	20.1 (17.8–22.5)	14.3 (12.4–16.5)	9.2 (8.6–9.9)	12.3 (11.1–13.6)	25.1 (23.2–27.1)
Unimproved	21.4 (19.4–23.6)	17.5 (15.1–20.3)	9.6 (8.6–10.8)	13.4 (12.0–15.0)	29.1 (23.7–35.0)
**Age of child (in months)**					
12–35	24.6 (22.7–26.6)	19.5 (17.3–21.9)	11.9 (11.1–12.6)	15.4 (14.1–16.7)	29.9 (27.5–32.4)
36–59	8.3 (6.7–10.3)	10.3 (8.5–12.5)	5.2 (4.6–5.8)	6.7 (5.6–8.0)	16.9 (14.5–19.7)
**Sex of child**					
Male	21.9 (19.7–24.3)	18.2 (15.8–20.9)	9.6 (8.9–10.3)	12.6 (11.5–13.8)	25.7 (22.9–28.8)
Female	19.9 (18.1–21.9)	13.6 (11.7–15.8)	9.0 (8.3–9.8)	13.0 (11.8–14.3)	25.7 (23.0–28.5)
**Birth size of child**					
Average or larger	21.0 (19.3–22.8)	15.6 (13.7–17.6)	8.6 (8.1–9.2)	12.1 (11.2–13.2)	24.0 (21.8–26.3)
Smaller than average	20.4 (17.0–24.4)	16.9 (14.4–19.7)	11.7 (10.6–13.0)	16.6 (14.4–18.9)	32.8 (28.4–37.5)
**Literacy status of mother**					
Illiterate	21.2 (17.9–25.1)	17.3 (15.3–19.5)	9.6 (8.8–10.4)	14.9 (13.4–16.6)	26.3 (23.8–29.0)
Literate	20.8 (19.1–22.7)	13.1 (10.9–15.7)	9.1 (8.4–9.8)	10.8 (9.7–12.0)	24.8 (22.1–27.8)
**Mother exposed to mass media**					
Not exposed	20.6 (18.5–22.9)	16.4 (14.1–19.0)	9.8 (8.8–10.8)	15.9 (14.0–18.0)	27.5 (23.9–31.4)
Exposed	21.1 (19.1–23.3)	15.7 (13.7–17.9)	9.1 (8.5–9.7)	11.3 (10.3–12.4)	24.9 (22.7–27.3)
**Religion**^2^					
Main	19.0 (16.9–21.4)	16.6 (14.7–18.7)	8.9 (8.3–9.5)	14.9 (13.5–16.5)	NC
Other	23.4 (21.3–25.6)	14.7 (11.8–18.2)	11.0 (9.8–12.2)	9.6 (8.5–10.8)	NC
**Caste/Tribe**					
Scheduled Caste	NA	NA	9.2 (8.2–10.5)	NA	NA
Scheduled Tribe	NA	NA	10.1 (8.6–11.8)	NA	NA
Other	NA	NA	9.3 (8.6–10.0)	NA	NA
**Wealth status**					
Poor	22.5 (19.9–25.5)	16.4 (14.1–18.9)	10.1 (9.3–11.0)	15.4 (13.7–17.3)	27.3 (24.4–30.4)
Middle	19.0 (16.5–21.9)	16.3 (13.7–19.2)	8.9 (8.1–9.9)	12.3 (11.0–13.7)	28.2 (25.4–31.3)
Rich	21.4 (19.3–23.8)	14.4 (11.2–18.4)	8.0 (7.2–9.0)	9.6 (8.1–11.4)	20.2 (17.3–23.4)
**Place of residence**					
Urban	22.3 (20.0–24.9)	13.2 (9.9–17.3)	9.0 (8.1–10.0)	11.4 (9.9–13.0)	25.5 (22.6–28.7)
Rural	20.2 (18.1–22.5)	16.5 (14.7–18.5)	9.4 (8.8–10.1)	13.6 (12.3–15.0)	25.8 (23.4–28.3)
**Total**	**20.9 (19.3–22.6)**	**16.0 (14.3–17.8)**	**9.3 (8.8–9.9)**	**12.8 (11.8–13.9)**	**25.7 (23.8–27.7)**

Abbreviations: CI, confidence interval; NA, Not applicable, NC: not collected.

All values are weighted.

^1^ estimated per 100 children.

^2^ termed as main if followed in the majority of households in the particular country (Christian in Democratic Republic of Congo and Ethiopia, Hindu in India and Islam in Nigeria).

Table 3 shows the regression results for ARI adjusted for various socioeconomic, demographic and residence related variables. Measles vaccination was statistically associated with lower prevalence of ARI in India and Pakistan. Measles-vaccinated children in India were only 0.85 (AOR: 0.85; 95% CI: 0.75, 0.96) times as likely as measles-unvaccinated children to suffer from ARI. Likewise, measles-vaccinated children in Pakistan were only 0.70 (AOR: 0.70; 95% CI: 0.59, 0.84) times as likely as measles-unvaccinated children to suffer from ARI. The variables that were statistically associated with ARI in the selected countries are type of cooking fuel, age and birth size of the child, mother’s literacy, religion, caste/tribe and household wealth.

**Table 3:** Results of multivariable binary logistic regression examining the effect of measles vaccination on ARI in children age 12–59 months in selected countries.

Socioeconomic, demographic and residence related characteristics	AOR (95% CI)				
	Democratic Republic of Congo	Ethiopia	India	Nigeria	Pakistan
	2013–14	2011	2005–06	2013	2012–13
	n = 6945	n = 5008	n = 24658	n = 12279	n = 4943
**Measles vaccination**					
Unvaccinated	Ref.	Ref.	Ref.	Ref.	Ref.
Vaccinated	0.90 (0.73–1.10)	0.97 (0.78–1.20)	**0.85 (0.75–0.96)**	1.03 (0.79–1.34)	**0.70 (0.59–0.84)**
**Type of cooking fuel**					
Other	Ref.	Ref.	Ref.	Ref.	Ref.
Solid	5.72 (0.79–41.5)	1.41 (0.55–3.64)	**1.38 (1.17–1.64)**	**2.11 (1.32–3.36)**	1.10 (0.90–1.35)
**Age of child (in months)**					
36–59	Ref.	Ref.	Ref.	Ref.	Ref.
12–35	**1.61 (1.25–2.07)**	1.24 (0.99–1.55)	**1.27 (1.13–1.43)**	**2.29 (1.65–3.19)**	**1.27 (1.08–1.49)**
**Sex of child**					
Female	Ref.	Ref.	Ref.	Ref.	Ref.
Male	1.08 (0.90–1.30)	1.04 (0.85–1.27)	1.03 (0.93–1.16)	1.03 (0.82–1.29)	1.09 (0.94–1.27)
**Birth size of child**					
Average or larger	Ref.	Ref.	Ref.	Ref.	Ref.
Smaller than average	1.01 (0.76–1.33)	1.23 (0.99–1.52)	**1.29 (1.14–1.47)**	1.13 (0.83–1.52)	**1.27 (1.06–1.52)**
**Literacy status of mother**					
Illiterate	Ref.	Ref.	Ref.	Ref.	Ref.
Literate	0.96 (0.76–1.21)	0.77 (0.60–1.00)	**1.17 (1.02–1.34)**	**1.56 (1.15–2.13)**	1.06 (0.88–1.27)
**Mother exposed to mass media**					
Not exposed	Ref.	Ref.	Ref.	Ref.	Ref.
Exposed	1.19 (0.97–1.47)	1.25 (1.00–1.55)	0.90 (0.78–1.05)	0.90 (0.69–1.17)	1.00 (0.83–1.20)
**Religion**^1^					
Main	Ref.	Ref.	Ref.	Ref.	NC
Other	0.86 (0.71–1.04)	0.94 (0.76–1.16)	**1.21 (1.08–1.38)**	0.85 (0.64–1.13)	NC
**Caste/Tribe**					
Other	NA	NA	Ref.	NA	NA
Scheduled Caste	NA	NA	1.07 (0.93–1.23)	NA	NA
Scheduled Tribe	NA	NA	**0.56 (0.47–0.68)**	NA	NA
**Wealth status**					
Rich	Ref.	Ref.	Ref.	Ref.	Ref.
Poor	1.01 (0.77–1.33)	1.34 (0.89–2.03)	**1.34 (1.10–1.64)**	**1.99 (1.33–2.98)**	**1.38 (1.07–1.78)**
Middle	1.12 (0.87–1.43)	1.42 (0.97–2.09)	**1.20 (1.02–1.42)**	1.45 (1.00–2.10)	**1.34 (1.09–1.65)**
**Place of residence**					
Urban	Ref.	Ref.	Ref.	Ref.	Ref.
Rural	1.06 (0.83–1.35)	1.27 (0.85–1.89)	0.88 (0.76–1.01)	0.87 (0.65–1.17)	1.00 (0.83–1.19)

Abbreviations: AOR, adjusted odds ratio; CI, confidence interval; NA, not applicable; NC, not collected.

Highlighted AOR indicates significant findings at p-value < 0.05.

^1^ termed as main if followed in the majority of households in the particular country (Christian in Democratic Republic of Congo and Ethiopia, Hindu in India and Islam in Nigeria).

Regression results adjusted for various socioeconomic, demographic and residence related variables suggest a significant statistical association between measles vaccination and lower prevalence of diarrhea in the Democratic Republic of Congo, India, Nigeria and Pakistan (Table 4). The measles-vaccinated children in these countries were statistically less likely to suffer from diarrhea compared to children who were not given the measles vaccine. The odds ratios ranged between 0.78 (in Democratic Republic of Congo) and 0.88 (in India). The other variables that were statistically associated with diarrhea were the type of toilet facility, age, sex and birth size of child, mother’s literacy and exposure to mass media, religion, household wealth, and place of residence.

**Table 4:** Results of multivariable binary logistic regression examining the effect of measles vaccination on diarrhea in children age 12–59 months in selected countries.

Socioeconomic, demographic and residence related characteristics	AOR (95% CI)				
	Democratic Republic of Congo	Ethiopia	India	Nigeria	Pakistan
	2013–14	2011	2005–06	2013	2012–13
	n = 6948	n = 5014	n = 24685	n = 12331	n = 4960
**Measles vaccination**					
Unvaccinated	Ref.	Ref.	Ref.	Ref.	Ref.
Vaccinated	**0.78 (0.68–0.89)**	0.89 (0.77–1.05)	**0.88 (0.81–0.99)**	**0.79 (0.70–0.90)**	**0.81 (0.69–0.96)**
**Type of toilet Facility**					
Improved and not shared	Ref.	Ref.	Ref.	Ref.	Ref.
Unimproved	**1.33 (1.13–1.56)**	1.20 (0.91–1.58)	1.10 (0.99–1.23)	0.93 (0.83–1.05)	1.14 (0.98–1.33)
**Source of Drinking water**					
Improved	Ref.	Ref.	Ref.	Ref.	Ref.
Unimproved	1.02 (0.89–1.17)	1.08 (0.92–1.27)	0.97 (0.87–1.08)	1.09 (0.97–1.22)	1.08 (0.90–1.28)
**Age of child (in months)**					
36–59	Ref.	Ref.	Ref.	Ref.	Ref.
12–35	**2.90 (2.43–3.47)**	**2.31 (1.93–2.75)**	**2.25 (2.03–2.49)**	**2.31 (2.00–2.68)**	**2.07 (1.78–2.42)**
**Sex of child**					
Female	Ref.	Ref.	Ref.	Ref.	Ref.
Male	1.09 (0.97–1.23)	**1.24 (1.07–1.43)**	**1.14 (1.04–1.24)**	0.93 (0.84–1.04)	**1.15 (1.01–1.32)**
**Birth size of child**					
Average or larger	Ref.	Ref.	Ref.	Ref.	Ref.
Smaller than average	1.14 (0.95–1.35)	1.10 (0.94–1.29)	**1.38 (1.25–1.52)**	**1.34 (1.16–1.54)**	**1.39 (1.19–1.64)**
**Literacy status of mother**					
Illiterate	Ref.	Ref.	Ref.	Ref.	Ref.
Literate	0.93 (0.80–1.09)	0.90 (0.76–1.08)	1.09 (0.98–1.21)	**1.17 (1.01–1.36)**	1.18 (1.00–1.38)
**Mother exposed to mass media**					
Not exposed	Ref.	Ref.	Ref.	Ref.	Ref.
Exposed	1.08 (0.95–1.24)	1.08 (0.92–1.26)	1.08 (0.96–1.22)	**0.82 (0.72–0.93)**	1.04 (0.88–1.23)
**Religion**^1^					
Main	Ref.	Ref.	Ref.	Ref.	NC
Other	**1.20 (1.06–1.35)**	0.88 (0.76–1.02)	**1.19 (1.08–1.32)**	**0.63 (0.55–0.72)**	NC
**Caste/Tribe**					
Other	NA	NA	Ref.	NA	NA
Scheduled Caste	NA	NA	1.00 (0.89–1.13)	NA	NA
Scheduled Tribe	NA	NA	0.96 (0.85–1.09)	NA	NA
**Wealth status**					
Rich	Ref.	Ref.	Ref.	Ref.	Ref.
Poor	1.00 (0.83–1.19)	1.03 (0.77–1.36)	**1.30 (1.12–1.51)**	**1.55 (1.28–1.87)**	**1.60 (1.29–2.00)**
Middle	0.94 (0.80–1.11)	1.06 (0.82–1.38)	**1.18 (1.04–1.34)**	**1.29 (1.09–1.52)**	**1.54 (1.29–1.85)**
**Place of residence**					
Urban	Ref.	Ref.	Ref.	Ref.	Ref.
Rural	0.93 (0.79–1.09)	1.30 (0.99–1.71)	0.92 (0.83–1.02)	**0.83 (0.72–0.96)**	0.96 (0.83–1.12)

Abbreviations: AOR, adjusted odds ratio; CI, confidence interval; NA, not applicable; NC, not collected.

Highlighted AOR indicates significant findings at p-value < 0.05.

^1^ termed as main if followed in the majority of households in the particular country (Christian in Democratic Republic of Congo and Ethiopia, Hindu in India and Islam in Nigeria).

Having found a plausible statistical association between measles vaccination and ARI in India and Pakistan, we estimated the VE in these countries. The VE indicated that measles vaccination was associated with a reduction in ARI by 15 percent in India (VE: 15%; 95% CI: 4–25%) and 30 percent in Pakistan (VE: 30%; 95% CI: 16–41%) in vaccinated children than in unvaccinated children. Having established a significant statistical association between measles vaccination and diarrhea in the Democratic Republic of Congo, India, Nigeria and Pakistan, we estimated the VE in these countries. Measles vaccination reduced diarrhea by 22 percent in the Democratic Republic of Congo (VE: 22%; 95% CI: 11–32%), 12 percent in India (VE: 12%; 95% CI: 1–19%), 21 percent in Nigeria (VE: 21%; 95% CI: 10–30%) and 19 percent in Pakistan (VE: 19%; 95% CI: 4–31%).

## Discussion

Our study is, perhaps, the first to examine the effect of measles vaccination on ARI and diarrhea in children age 12–59 months. The study uses the most recent round of DHS in the selected countries for this purpose. The measles vaccinated children were on an average two months older than the measles unvaccinated children. The coverage of measles vaccination was above 60 percent in the Democratic Republic of Congo, Ethiopia, India and Pakistan. The prevalence of ARI and diarrhea varied significantly across the five countries. The prevalence of ARI ranged between 2 percent in Nigeria and 18 percent in Pakistan. Likewise, the prevalence of diarrhea ranged between 9 percent in India and 26 percent in Pakistan. The prevalence of ARI and diarrhea also varied considerably by socioeconomic, demographic and residence related variables.

The receipt of measles vaccination was associated with decrease in ARI in India and Pakistan. Measles-vaccinated children were statistically less likely to suffer from ARI than those who were not given the measles vaccine. Measles vaccination was associated with a reduction of ARI in vaccinated children by 15 percent in India and 30 percent in Pakistan. Measles vaccination also provided a protective effect against diarrhea in four out of the five countries considered in the analysis—the Democratic Republic of Congo, India, Nigeria and Pakistan. Children who were given the measles vaccine had a lower risk of diarrhea than their counterparts who had not been given the vaccine. Compared to unvaccinated children, measles vaccination was associated in reducing diarrhea in vaccinated children by 22 percent in the Democratic Republic of Congo, 12 percent in India, 21 percent in Nigeria and 19 percent in Pakistan. Our findings are consistent with some small-scale studies conducted in Bangladesh, Brazil, India and Zimbabwe [20–24]. Strikingly, measles vaccination works as a preventive measure against pneumonia and diarrhea, only if both diseases occur either as a measles complication or secondary infection. Besides, there are several other causative pathogens of pneumonia and diarrhea and those pathogens have a higher incidence than measles associated pneumonia and diarrhea. The most common pathogens causing pneumonia are *Streptococcus pneumoniae* (pneumococcus) and *Heamophilus influenzae type b* (Hib) and that causing diarrhea is Rotavirus. Besides, the burden of these other pathogens varies across the countries [19,44]. Because of these, we may not have found the protective effect of measles vaccination on ARI in the Democratic Republic of Congo, Ethiopia and Nigeria and on diarrhea in Ethiopia.

Household environment factors were also statistically associated with ARI and diarrhea. The risk of ARI was higher in children belonging to households using solid cooking fuels than those belonging to households using other cooking fuels in India and Nigeria. Households’ access to improved toilet facilities decreased the risk of diarrhea in children in the Democratic Republic of Congo. These findings are consistent with several past studies on this subject [35,45–48]. Age and birth size of child, mother’s literacy, religion and household wealth were statistically associated with ARI and diarrhea in children. Caste/tribe was also statistically associated with ARI. Similarly, diarrhea was statistically associated with the sex of the child, mother’s exposure to media and place of residence. Many previous studies in low- and middle-income countries support our findings [22,32–40].

In 2008, the WHO Strategic Advisory Group of Experts (SAGE) on immunization recommended that every child should get two doses of measles vaccine. The first dose should be given through the routine immunization schedule and the second dose could be given through either the routine immunization schedule or supplementary immunization activities (SIAs) [49–51]. All 47 countries having highest burden of measles (including the Democratic Republic of Congo, Ethiopia, India Nigeria and Pakistan) have introduced a second dose of measles vaccine in their immunization program [52,53]. At present, the routine immunization schedule in both India and Pakistan includes two doses of measles vaccine. In the Democratic Republic of Congo, Ethiopia and Nigeria, the first dose is given through the routine immunization schedule and the second dose is given through SIA [54,55].

In addition to SAGE recommendations on measles vaccination, the WHO and UNICEF have included measles vaccination in GAPPD as a preventive intervention for pneumonia and diarrhea [19]. Our findings based on large-scale country representative household surveys lend support to the WHO and UNICEF initiative. Also we found among measles unvaccinated children, only 13% in the Democratic Republic of Congo and India, 9% in Ethiopia and Pakistan, and 5% in Nigeria received other childhood vaccines (including Bacillus Calmette-Guerin (BCG), 3 doses of Polio and 3 doses of Diphtheria Pertussis, and Tetanus (DPT) vaccine). Our study findings call for the promotion of childhood vaccination at the recommended age in low- and middle- income countries. Additionally, the measles vaccination campaigns must highlight the preventive benefit of measles vaccine against pneumonia and diarrhea. The immunization program must ensure that each child gets the two recommended doses of measles vaccine at the required ages. In case the child misses the first dose, s/he must get the second dose through either routine immunization program or SIAs.

### Strengths and Limitations

The strengths and limitations of the study must be noted. Our findings are robust and representative for the countries included in the analysis because we used large-scale nationally representative population-based DHS data to examine the effect of measles vaccination on ARI and diarrhea in children. Second, we could control for a number of socioeconomic, demographic and residence related variables in the regression models. However, there are a few limitations. One is the use of ARI as a proxy for pneumonia. Pneumonia is the most severe form of ARI affecting the lungs. Unfortunately, DHS does not collect direct information on pneumonia. Instead, DHS poses a series of questions on the symptoms of ARI. Second, we could not attribute the ARI and diarrhea cases to measles in children because DHS does not collect any information on the prevalence of measles. However, large measles epidemics occurred in the Democratic Republic of Congo, Ethiopia, Nigeria and Pakistan in 2010–13 [25,56–58]. In India, measles is endemic in nature, and 6 outbreaks were serologically confirmed by Field Epidemiology Training Program (FETP) in 2004–06 [59]. Third, we used birth size as a proxy for birth weight. The birth weight data was available for only 5 percent of the children in Ethiopia, 36 percent in India, 16 percent in Nigeria and 12 percent in Pakistan [27,28,30,31]. Fourth, we used cross-sectional datasets to establish the association of measles vaccination with ARI and diarrhea. Fifth, the prevalence of ARI and diarrhea were derived from the mother’s report in a 14-day recall period. However, it may be noted that all the DHS and UNICEF sponsored surveys conducted in various countries are cross-sectional in nature and use a 14-day recall period to determine the prevalence of ARI and diarrhea in children [60]. Owing to the unavailability of the most recent DHS data in India, we analysed NFHS-3 (2005–06) data. At present, NFHS-4 is in progress in India and the data is expected to be available by the end of 2017 [DHS is also known as National Family Health Survey (NFHS) in India]. Since our analysis is based on cross-sectional data, more analysis is needed to confirm the protective effect of measles vaccination on ARI and diarrhea.

## Conclusion

Our study demonstrates the protective effect of measles vaccination on ARI and diarrhea in children in five countries that report the highest number of child deaths from pneumonia and diarrhea. Measles vaccination has effectively reduced ARI and diarrhea cases in children. Thus, measles vaccination can play an important role in reducing mortality from pneumonia and diarrhea in children, particularly, in countries that have a high incidence of measles. Thus, the immunization program must ensure that the measles vaccine is administered at the recommended age to every child.

## Supporting Information

S1 Appendix Variable description file. (DOCX)
