# Supplementary material for: Does Measles Vaccination Reduce the Risk of Acute Respiratory Infection (ARI) and Diarrhea in Children: A Multi-Country Study?
Source: PLoS One. 2017 Jan 11;12(1):e0169713. doi: 10.1371/journal.pone.0169713 (PMC5226778; doi:10.1371/journal.pone.0169713)
Supplement: S1 Appendix — (DOCX) [file pone.0169713.s001.docx]

**S1 Appendix: Variable description file**

| **Sr. No.** | **Control variables** | **Category** |
| --- | --- | --- |
| 1 | Cooking fuel | 1. **Solid** (Coal, lignite, charcoal, wood, straw/shrubs/grass, agricultural crop and animal dung)  2. **Other** (Electricity, LPG, biogas and kerosene) |
| 2. | Type of toilet facility | 1. **Improved and not shared** (Flush toilet to piped sewer system, to septic tank or to pit latrine; Pit toilet latrine ventilated improved pit (VIP) or with slab and Composting toilet)  2. **Unimproved** (Flush to somewhere else or don’t know where; Pit latrine without slab/open pit; No facility/uses bush/field; Dry toilet; Other) |
| 3. | Source of drinking water | 1. **Improved** (Piped water into dwelling or to yard/plot; Public tap/standpipe; Tube well or borehole; Protected well; Protected spring and Bottled water; Rain water)  2. **Unimproved** (Unprotected dug well; Unprotected well; Unprotected spring; River/dam/lake/ponds/stream/canal/irrigation channel; Tanker truck; Cart with small tank; Other) |
| 4 | Age of child (in months) | 1. 12-35  2. 36-59 |
| 5 | Sex of child | 1. Male  2. Female |
| 6 | Size of child at birth | 1. Average or larger  2. Smaller than average |
| 7 | Literacy status of mother | 1. Illiterate  2. Literate |
| 8 | Mother exposed to mass media | 1. Not exposed (Neither watch TV nor listen radio nor read magazine)  2. Exposed to (Either watch TV or listen radio or read magazine) |
| 9 | Religion^1^ | 1. Main  2. Other |
| 10 | Caste^2^ | 1. Scheduled Caste (SC)  2. Scheduled Tribe (ST)  3. Other |
| 11 | Wealth status | 1. Poor  2. Middle  3. Rich |
| 12 | Place of residence | 1. Urban  2. Rural |

^1^ termed as main if followed in the majority of households in the particular country (Christian in Democratic Republic of Congo and Ethiopia, Hindu in India and Islam in Nigeria).

^2^ Included as control variable only in the analysis for India.
